# Which Prostate Cancers are Undetected by Multiparametric Magnetic Resonance Imaging in Men with Previous Prostate Biopsy? An Analysis from the PICTURE Study

**DOI:** 10.1016/j.euros.2021.06.003

**Published:** 2021-06-15

**Authors:** Joseph M. Norris, Lucy A.M. Simmons, Abi Kanthabalan, Alex Freeman, Neil McCartan, Caroline M. Moore, Shonit Punwani, Hayley C. Whitaker, Mark Emberton, Hashim U. Ahmed

**Affiliations:** a UCL Division of Surgery & Interventional Science, University College London, London, UK; b Department of Urology, University College London Hospitals NHS Foundation Trust, London, UK; c Department of Urology, North Bristol NHS Trust, Bristol, UK; d Department of Urology, North West Anglia NHS Foundation Trust, Peterborough, UK; e Department of Pathology, University College London Hospitals NHS Foundation Trust, London, UK; f Department of Radiology, University College London Hospitals NHS Foundation Trust, London, UK; g Department of Urology, Imperial College Healthcare NHS Trust, London, UK; h Imperial Prostate, Department of Surgery & Cancer, Faculty of Medicine, Imperial College London, London, UK

**Keywords:** False-negative magnetic resonance imaging, Undetected cancer, Multiparametric magnetic resonance imaging, PICTURE study, Prostate cancer

## Abstract

**Background:**

Multiparametric magnetic resonance imaging (mpMRI) has improved risk stratification for suspected prostate cancer in patients following prior biopsy. However, not all significant cancers are detected by mpMRI. The PICTURE study provides the ideal opportunity to investigate cancer undetected by mpMRI owing to the use of 5 mm transperineal template mapping (TTPM) biopsy.

**Objective:**

To summarise attributes of cancers systematically undetected by mpMRI in patients with prior biopsy.

**Design, setting, and participants:**

PICTURE was a paired-cohort confirmatory study in which men requiring repeat biopsy underwent mpMRI followed by TTPM biopsy.

**Outcome measurements and statistical analysis:**

Attributes were compared between cancers detected and undetected by mpMRI at the patient level. Four predefined histopathological thresholds were used as the target condition for TTPM biopsy. Application of prostate-specific antigen density (PSAD) was explored.

**Results and limitations:**

When nonsuspicious mpMRI was defined as Likert score 1–2, 2.9% of patients (3/103; 95% confidence interval [CI] 0.6–8.3%) with definition 1 disease (Gleason ≥ 4 + 3 of any length or maximum cancer core length [MCCL] ≥ 6 mm of any grade) had their cancer not detected by mpMRI. This proportion was 6.5% (11/168; 95% CI 3.3–11%) for definition 2 disease (Gleason ≥ 3 + 4 of any length or MCCL ≥ 4 mm of any grade), 4.8% (7/146; 95% CI 2.0–9.6%) for any amount of Gleason ≥ 3 + 4 cancer, and 9.3% (20/215; 95% CI 5.8–14%) for any cancer. Definition 1 cancers undetected by mpMRI had lower overall Gleason score (*p* = 0.02) and maximum Gleason score (*p* = 0.01) compared to cancers detected by mpMRI. Prostate cancers undetected by mpMRI had shorter MCCL than cancers detected by mpMRI for every cancer threshold: definition 1, 6 versus 8 mm (*p* = 0.02); definition 2, 5 versus 6 mm (*p* = 0.04); any Gleason ≥ 3 + 4, 5 versus 6 mm (*p* = 0.03); and any cancer, 3 versus 5 mm (*p* = 0.0009). A theoretical PSAD threshold of 0.15 ng/ml/ml reduced the proportion of patients with undetected disease on nonsuspicious mpMRI to 0% (0/105; 95% CI 0–3.5%) for definition 1, 0.58% (1/171; 95% CI 0.01–3.2%) for definition 2, and 0% (0/146) for any Gleason ≥ 3 + 4.

**Conclusions:**

Few significant cancers are undetected by mpMRI in patients requiring repeat prostate biopsy. Undetected tumours are of lower overall and maximum Gleason grade and shorter cancer length compared to cancers detected by mpMRI.

**Patient summary:**

In patients with a previous prostate biopsy, magnetic resonance imaging (MRI) overlooks few prostate cancers, and these tend to be smaller and less aggressive than cancer that is detected.

## Take Home Message

In men with previous prostate biopsy, few prostate cancers are not detected by multiparametric magnetic resonance imaging (mpMRI), and these are lower in size and grade than prostate cancer that is detected by mpMRI. This further reinforces the role that mpMRI should play in assessment of suspected prostate cancer.

## 1 Introduction

Prebiopsy multiparametric magnetic resonance imaging (mpMRI) has excellent test accuracy, validity, and reliability for detection of clinically significant prostate cancer [1], [2], [3], [4], [5] resulting in its incorporation into national and international guidelines [6], [7]. However, as with all cancer risk-stratification strategies, not every prostate cancer is detected by mpMRI [1]. Understanding the nature of disease that is undetected by mpMRI is important, particularly given the increasing preference for omission of prostate biopsy in cases of nonsuspicious prebiopsy imaging [6]. We have recently shown that in biopsy-naïve patients, so-called mpMRI-invisible cancer is significantly smaller in tumour size and has lower maximum and overall Gleason scores compared to mpMRI-visible disease [8].

Recent investigation into mpMRI performance in patients with prior biopsy has shown favourable features of undetected disease [9], consistent with a body of evidence identifying reassuring genetic, molecular, histopathological, and clinical characteristics for mpMRI-undetected cancer in biopsy-naïve patients [8], [10], [11], [12]. Nonetheless, concern remains regarding the potential for significant prostate cancer going undetected on mpMRI [13]. Existing evidence for men with prior biopsy is limited by imperfect reference standards, retrospective study designs, lower mpMRI magnetic strength, or poor image quality due to close timing between prior biopsy and imaging [14], [15], [16], [17].

The Prostate Imaging Compared to Transperineal Ultrasound-guided biopsy for significant prostate cancer Risk Evaluation (PICTURE) study was a prospective paired-cohort confirmatory study that compared the diagnostic performance of mpMRI against a strict reference standard in 249 patients with prior prostate biopsy who required further risk stratification [3], [18], [19], [20]. Patients underwent prebiopsy mpMRI at 3T, followed by transperineal template prostate mapping (TTPM) biopsy (the reference test) in which biopsies were taken at 5 mm intervals throughout the prostate. Here we present a comparison of cancer attributes (at the patient level) between patients with mpMRI-detected and mpMRI-undetected disease in the PICTURE study.

## 2 Patients and methods

### 2.1 Study population

**Fig. 1:**
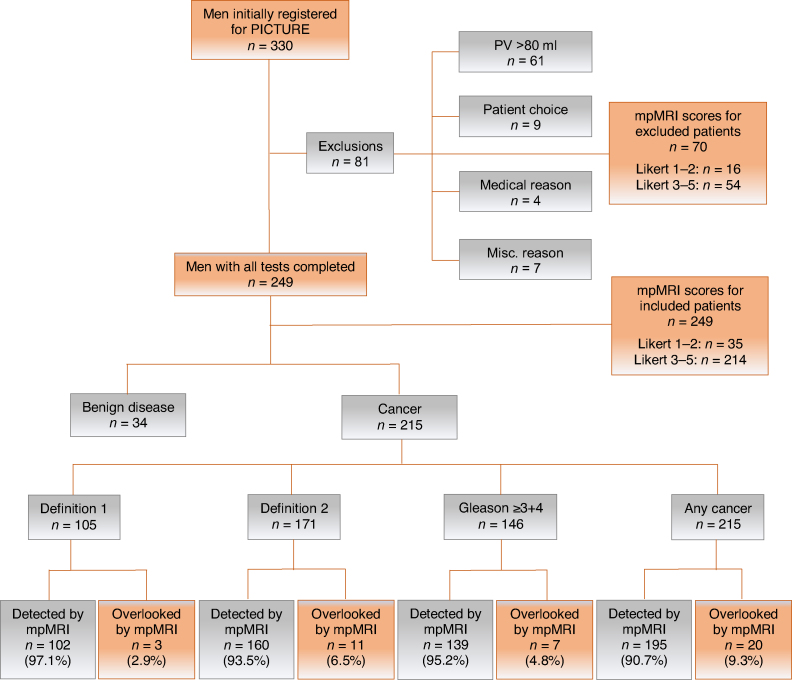
Flow chart for study inclusion. Misc. = miscellaneous; mpMRI = multiparametric magnetic resonance imaging; PV = prostate volume. Nondetection on mpMRI was defined as Likert score 1–2.

In brief, PICTURE was a prospective single-centre trial in which patients with prior systematic transrectal ultrasound (TRUS)-guided biopsy and ongoing clinical suspicion underwent prebiopsy 3T mpMRI, followed by TTPM biopsy under general anaesthesia. The mpMRI parameters used are reported in full in the main PICTURE report [3]. Each test was performed and reported blinded to results. Patients remained blinded to mpMRI results. PICTURE was registered on ClinicalTrials.gov (NCT01492270). The study protocol for PICTURE has been described in detail elsewhere [3], [18]. Ethics committee approval for PICTURE was granted by London City Road and Hampstead National Research Ethics Committee (11/LO/1657). For the present study, all patients with prostate cancer were included (Fig. 1).

### 2.2 Definitions of clinical significance

Three thresholds for prostate cancer on TTPM biopsy were defined as the target conditions of interest to incorporate and reflect the uncertainty about what constitutes clinically significant prostate cancer. PROMIS study definition 1 was overall Gleason score ≥ 4 + 3 of any length or a maximum cancer core length (MCCL) ≥ 6 mm of any grade. PROMIS definition 2 was overall Gleason score ≥ 3 + 4 of any length or MCCL ≥ 4 mm of any grade. These two criteria were developed and validated for TTPM biopsy for the detection of Gleason score 4 [21] and cancer core lengths representative of lesions of 0.5 ml and 0.2 ml [22], [23], [24], [25] and were used in the main PICTURE and PROMIS trials [1], [3]. The third threshold for clinically significant disease was any amount of overall Gleason score ≥ 3 + 4 cancer. The presence of any cancer was also used for completion.

### 2.3 Post hoc analysis

Once stratified by cancer threshold, patients were divided into mpMRI-detected (Likert score 3–5) and mpMRI-undetected (Likert score 1–2) groups. An additional threshold for tumour visibility was also evaluated (mpMRI-detected group, Likert score 4–5; mpMRI-undetected group, Likert score 1–3). Prostate-specific antigen density (PSAD) was calculated by dividing serum PSA by mpMRI-derived prostate volume (using the prolate ellipsoid method). Overall Gleason score was defined as the predominant Gleason pattern across the entire prostate and constituted the final pathological score. Maximum Gleason score was defined as the highest Gleason pattern found per patient.

### 2.4 Statistical analysis

We described the characteristics for the mpMRI-detected and mpMRI-undetected cancer groups and then stratified analysis according to the four cancer thresholds. Mean values with standard deviation and median values with interquartile range (IQR) were calculated with descriptive statistical techniques to characterise the measures of central tendency for demographic patient data, MCCL measurements, and PSAD values. Data distribution was evaluated using D’Agostino-Pearson or Shapiro-Wilk normality tests. All outcome data were unpaired and had a non-normal distribution, so two-sided nonparametric statistical tests were used. At the patient level, overall and maximum Gleason scores were compared using the χ^2^ test, whilst MCCL and PSAD values were compared using the Mann-Whitney U test. The α level was 0.05 for all statistical tests. Binomial 95% confidence intervals (CIs) for proportions were calculated via approximation with the Poisson distribution method. Multiple testing was assessed via the false discovery rate using the Benjamini-Hochberg method. All analyses were conducted using GraphPad Prism 9.0.0 (GraphPad Software, La Jolla, CA, USA) and the R statistical environment (v3.6.3; R Foundation for Statistical Computing, Vienna, Austria).

## 3 Results

### 3.1 Overall detection

Demographic data for all 249 patients included in the final PICTURE analysis are shown in Table 1. When nonsuspicious mpMRI was defined as Likert score 1–2, 2.9% (3/103; 95% CI 0.6–8.3%) of patients with definition 1 disease had their cancer undetected by mpMRI. This proportion was 6.5% (11/168; 95% CI 3.3–11%) for definition 2 disease, 4.8% (7/146; 95% CI 2.0–9.6%) for any amount of Gleason ≥ 3 + 4 cancer, and 9.3% (20/215; 95% CI 5.8–14%) for any cancer. When nonsuspicious mpMRI was defined as Likert score 1–3, 19% (20/103; 95% CI 12–28%) with definition 1 disease, 32% (54/168; 95% CI 25–40%) with definition 2 disease, 30% (44/146; 95% CI 23–38%) with any Gleason ≥ 3 + 4, and 41% (89/215; 95% CI 35–48%) with any cancer had their cancer undetected by mpMRI.

**Table 1:** Summary of demographic data for all patients within PICTURE

Parameter	Result
Sample size (*n*)	249
Mean age, yr (standard deviation)	62.0 (7.2)
Median prostate-specific antigen, ng/ml (IQR)	6.8 (4.8–9.8)
Median prostate volume, ml (IQR)	37.0 (26.8–50.0)
Family history of prostate cancer, *n* (%)	78 (31)
Ethnicity, *n* (%)	
White	208 (84)
Black	25 (10)
Asian	8 (3)
Hispanic	1 (0.4)
Other	5 (2)
Median time since previous biopsy, d (IQR)	386 (269–607)
Median number of previous biopsies per patient, *n* (IQR)	12 (11–13)
Median number of cores taken per previous biopsy, *n* (IQR)	1 (1–2)
Previous biopsy description, *n* (%)	
Transrectal ultrasound biopsy	342 (98)
Transperineal template mapping biopsy	6 (1.7)
Positive pathology result	217 (62)
Negative pathology result	127 (36)
Pathology report unavailable	4 (1.1)
Histopathology on previous biopsy, *n* (%)	
No cancer	74 (30)
Gleason 2 + 3	2 (0.8)
Gleason 3 + 3	121 (49)
Gleason 3 + 4	48 (19)
Gleason 4 + 3	4 (1.6)
Likert score on multiparametric magnetic resonance imaging, *n* (%)	
1	1 (0.4)
2	34 (14)
3	85 (34)
4	55 (22)
5	74 (30)
Median prostate-specific antigen density, ng/ml/ml (IQR)	0.18 (0.12–0.28)
Overall Gleason score on transperineal template mapping biopsy, *n* (%)	
3 + 3	69 (32)
3 + 4	112 (52)
3 + 5	1 (0.47)
4 + 3	29 (13)
4 + 4	3 (1.4)
5 + 4	1 (0.47)
MCCL on transperineal template mapping biopsy, *n* (%)	
1–5 mm	119 (55)
6–10 mm	79 (37)
11–15 mm	17 (7.9)
Median MCCL on transperineal template mapping biopsy, mm (IQR)	5 (3–8)

IQR = interquartile range; MCCL = maximum cancer core length.

### 3.2 Cancer grade

Table 2 compares key pathological outcomes between mpMRI-detected and mpMRI-undetected prostate cancer. Definition 1 cancers undetected by mpMRI had lower overall Gleason scores (*p* = 0.02) and maximum Gleason scores (*p* = 0.01) compared to cancers detected by mpMRI; this was also the case when evaluating any cancer (*p* = 0.01 and *p* = 0.02, respectively).

**Table 2:** Comparison of key histopathological outcomes at the patient level for mpMRI-detected and mpMRI-undetected prostate cancer in PICTURE for all four cancer thresholds on transperineal template mapping biopsy a

Characteristic	Cancer definition 1			Cancer definition 2			Gleason ≥ 3 + 4			Any cancer		
	PCaD	PCaU	Difference (95% CI)	PCaD	PCaU	Difference (95% CI)	PCaD	PCaU	Difference (95% CI)	PCaD	PCaU	Difference (95% CI)
Sample size, % (*n*/*N*)	97 (102/105)	2.9 (3/105)	94.1 (90–99)	94 (160/171)	6.4 (11/171)	87.6 (82–92)	95 (139/146)	4.8 (7/146)	90.2 (86–95)	91 (195/215)	9.3 (20/215)	81.7 (76–87)
Overall GS, % (*n*/*N*)			*p* = 0.02			*p* = 0.09			*p* = 0.1			*p* = 0.01
3 + 3	8.8 (9/102)	33 (1/3)	24.2 (−29 to 78)	13 (21/160)	36 (4/11)	23 (−5.7 to 52)	0 (0/139)	0 (0/7)	–	29 (56/195)	65 (13/20)	36 (14–58)
3 + 4	59 (60/102)	33 (1/3)	26 (−29 to 80)	66 (106/160)	55 (6/11)	11 (−19 to 42)	76 (106/139)	86 (6/7)	10 (−17 to 36)	54 (106/195)	30 (6/20)	24 (3.1–56)
3 + 5	0.98 (1/102)	0 (0/5)	–	0.63 (1/160)	0 (0/11)	–	0.72 (1/139)	0 (0/7)	–	14 (28/195)	0 (0/20)	–
4 + 3	27 (28/102)	33 (1/3)	6 (−48 to 60)	18 (28/160)	9.1 (1/11)	8.9 (−9.6 to 26)	20 (28/139)	14 (1/7)	6 (−21 to 33)	0.51 (1/195)	5 (1/20)	4.5 (−5.1 to 14)
4 + 4	2.9 (3/102)	0 (0/3)	–	1.9 (3/160)	0 (0/11)	–	2.2 (3/139)	0 (0/7)	–	1.5 (3/195)	0 (0/20)	–
5 + 4	0.98 (1/102)	0 (0/3)	–	0.63 (1/160)	0 (0/11)	–	0.72 (1/139)	0 (0/7)	–	0.51 (1/195)	0 (0/20)	–
Overall MCCL, % (*n*/*N*)			*p* = 0.02			*p* = 0.04			*p* = 0.03			*p* = 0.0009
1–5 mm	7.8 (8/102)	33 (1/3)	25.2 (−28 to 80)	41 (66/160)	82 (9/11)	41 (17–65)	39 (54/139)	86 (6/7)	47 (20–74)	52 (101/195)	90 (18/20)	38 (23–53)
6–10 mm	75 (77/102)	66 (2/3)	9 (−45 to 63)	48 (77/160)	18 (2/11)	30 (5.9–54)	49 (68/139)	14 (1/7)	35 (7.4–62)	39 (77/195)	10 (2/20)	29 (15–44)
11–15 mm	17 (17/102)	0 (0/3)	–	11 (17/160)	0 (0/11)	–	12 (17/139)	0 (0/7)	–	8.7 (17/195)	0 (0/20)	–
Median MCCL, mm (IQR)	8 (6–10)	6 (2–6)	2 (0–6)	6 (4–8)	5 (4–5)	1 (0–3)	6 (4–9)	5 (3–5)	1 (0–4)	5 (3–8)	3 (2–5)	2 (1–3)

CI = confidence interval; GS = Gleason score; IQR = interquartile range; MCCL = maximum cancer core length; PCaD = prostate cancer detected by mpMRI; PCaU = prostate cancer undetected by mpMRI.

a Nonsuspicious mpMRI defined as Likert score 1–2.

On a per-patient basis, no cancers with overall Gleason score > 4 + 3 (Gleason grade groups 4–5) on TTPM biopsy were undetected by mpMRI (95% CI 0–52%; Table 3). Furthermore, no cancer with maximum Gleason score > 4 + 3 (Gleason grade groups 4–5) on TTPM biopsy were undetected by mpMRI (95% CI 0–52%). No primary, secondary, or tertiary Gleason pattern 5 was undetected by mpMRI (95% CI 0–84%).

**Table 3:** Proportions of prostate cancers detected and not detected by mpMRI in PICTURE by Gleason grade group a

Grade group	mpMRI-detected cancer, % (*n*/*N*)	mpMRI-undetected cancer, % (*n*/*N*)	Difference, % (95% CI)
GG 1	29 (56/195)	65 (13/20)	−36 (−58 to −14)
GG 2	54 (106/195)	30 (6/20)	24 (3.1–46)
GG 3	14 (28/195)	5.0 (1/20)	9.0 (−1.4 to 20)
GG 4	2.0 (4/195)	0 (0/20)	–
GG 5	0.5 (1/195)	0 (0/20)	–

CI = confidence interval; GG = Gleason grade group; mpMRI = multiparametric magnetic resonance imaging.

a Nonsuspicious mpMRI defined as Likert score 1–2.

### 3.3 Cancer core length

Prostate cancers undetected by mpMRI had shorter MCCL than those detected by mpMRI for every cancer threshold: definition 1, 6 versus 8 mm (difference 2 mm, 95% CI 0–6; *p* = 0.02); definition 2, 5 versus 6 mm (difference 1 mm, 95% CI 0–3; *p* = 0.04); any Gleason ≥ 3 + 4 cancer, 5 versus 6 mm (difference 1 mm, 95% CI 0–4; *p* = 0.03); and any cancer, 3 versus 5 mm (difference 2 mm, 95% CI 1–3; *p* = 0.0009).

When nonsuspicious mpMRI was defined as Likert score 1–3, prostate cancers undetected by mpMRI had significantly shorter MCCL than prostate cancers detected by mpMRI for all cancer definitions: definition 1, 6 versus 8 mm (difference 2 mm, 95% CI 1–3; *p* = 0.0008); definition 2, 4.5 versus 7 mm (difference 2.5 mm, 95% CI 1–3; *p* < 0.0001); any Gleason ≥ 3 + 4 cancer, 4 versus 7 mm (difference 3 mm, 95% CI 2–4; *p* < 0.0001); and any cancer, 3 versus 6 mm (difference 3 mm, 95% CI 2–4; *p* <  0.0001).

### 3.4 PSAD

Overall, median PSAD was 0.18 ng/ml/ml (IQR 0.12–0.28) across the entire cohort. For men with prostate cancer, PSAD did not significantly differ between those with mpMRI-detected disease (Likert score 3–5) and those with mpMRI-undetected disease (Likert score 1–2). However, application of theoretical PSAD thresholds above which a biopsy would be indicated altered the rates of undetected significant prostate cancer. Multiple hypothetical PSAD thresholds were evaluated for all cancer definitions and mpMRI detection thresholds (Table 4).

**Table 4:** Proportions of men with mpMRI-detected and mpMRI-undetected prostate cancer by theoretical prostate-specific antigen density thresholds above which a biopsy would be indicated for nonsuspicious mpMRI

	Prostate-specific antigen density threshold							
	0.08	0.10	0.12	0.15	0.18	0.20	0.22	0.25
**Nonsuspicious mpMRI: Likert 1–2**								
mpMRI-detected disease, *n* (%)								
Definition 1 cancer	105 (100)	105 (100)	105 (100)	105 (100)	105 (100)	105 (100)	105 (100)	105 (100)
Definition 2 cancer	171 (100)	171 (100)	171 (100)	170 (99)	169 (99)	168 (98)	168 (98)	166 (97)
Gleason ≥ 3 + 4	146 (100)	146 (100)	146 (100)	146 (100)	145 (99)	144 (99)	144 (99)	143 (98)
mpMRI-undetected disease, *n* (%)								
Cancer definition 1	0 (0)	0 (0)	0 (0)	0 (0)	0 (0)	0 (0)	0 (0)	0 (0)
Cancer definition 2	0 (0)	0 (0)	0 (0)	1 (0.58)	2 (1.2)	3 (1.8)	3 (1.8)	5 (2.9)
Gleason ≥ 3 + 4	0 (0)	0 (0)	0 (0)	0 (0)	1 (0.68)	2 (1.4)	2 (1.4)	3 (2.0)
Biopsies avoided, *n* (%) a	2 (0.80)	5 (2.0)	10 (4.0)	14 (5.6)	23 (9.2)	24 (9.6)	28 (11)	29 (12)
**Nonsuspicious mpMRI: Likert 1–3**								
mpMRI-detected disease, *n* (%)								
Cancer definition 1	105 (100)	104 (99)	103 (98)	103 (98)	103 (98)	102 (97)	101 (96)	98 (93)
Cancer definition 2	169 (99)	167 (98)	164 (96)	159 (93)	155 (91)	151 (88)	148 (87)	141 (82)
Gleason ≥ 3 + 4	145 (99)	143 (98)	140 (96)	136 (93)	133 (91)	129 (88)	127 (87)	121 (83)
mpMRI-undetected disease, *n* (%)								
Cancer definition 1	0 (0)	1 (0.95)	2 (1.9)	2 (1.9)	2 (1.9)	3 (2.9)	4 (3.8)	7 (6.7)
Cancer definition 2	2 (1.2)	4 (2.3)	7 (4.1)	12 (7.0)	16 (9.4)	20 (12)	23 (13)	30 (18)
Gleason ≥ 3 + 4	1 (0.68)	3 (2.1)	6 (4.1)	10 (6.8)	13 (8.9)	17 (12)	19 (13)	25 (17)
Biopsies avoided biopsies, *n* (%) a	15 (6.0)	24 (9.6)	38 (15)	55 (22)	73 (29)	81 (33)	88 (35)	92 (37)

mpMRI = multiparametric magnetic resonance imaging.

a Numbers of biopsies avoided were derived by applying the prostate-specific antigen density threshold across the entire cohort (including men with no cancer).

When nonsuspicious mpMRI was defined as Likert score 1–2, a PSAD threshold of 0.15 ng/ml/ml reduced the proportion of patients with undetected disease to 0% (0/105; 95% CI 0–3.5%) for definition 1, 0.58% (1/171; 95% CI 0.01–3.2%) for definition 2, and 0% (0/146; 95%CI 0–2.5%) for any Gleason ≥ 3 + 4. A PSAD threshold of 0.10 ng/ml/ml also reduced the proportion of patients with undetected disease to 0% (0/105; 95% CI 0–3.5%), 0% (0/171; 95% CI 0–2.1%), and 0% (0/146; 95%CI 0–2.5%), respectively. However, when considering the entire cohort (including those with benign disease), the number of biopsies that could potentially be avoided decreased from 14% (35/249) when no PSAD threshold was applied to nonsuspicious mpMRI (Likert scores 1–2) to 5.6% (14/249) when a PSAD threshold of 0.15 ng/ml/ml was applied, and to 2.0% (5/249) for a PSAD threshold of 0.10 ng/ml/ml.

When nonsuspicious mpMRI was defined as Likert score 1–3, a PSAD threshold of 0.15 ng/ml/ml reduced the proportion of patients with undetected disease to 1.9% (2/105; 95% CI 0.23–6.7%) for definition 1, 7.0% (12/171; 95% CI 3.7–12%) for definition 2, and 6.8% (10/146; 95%CI 3.3–12%) for any Gleason ≥ 3 + 4. A PSAD threshold of 0.10 ng/ml/ml also lowered the proportion of patients with undetected disease to 0.95% (1/105; 95% CI 0.02–5.2%), 2.3% (4/171; 95% CI 0.64–5.9%), and 2.1% (3/146; 95% CI 0.43–5.9%), respectively. Again, the number of biopsies that could potentially be avoided across the entire cohort decreased from 48% (120/249) when no PSAD threshold was applied to nonsuspicious mpMRI (Likert score 1–3) to 22% (55/249) when a PSAD threshold of 0.15 ng/ml/ml was applied, and to 9.6% (24/249) for a PSAD threshold of 0.10 ng/ml/ml.

## 4 Discussion

In summary, our post hoc analysis of the PICTURE cohort showed that for patients with previous TRUS-guided biopsy, the proportion of the most aggressive prostate tumours undetected by 3T mpMRI is very low (2.9%). Overall, our findings in this patient subgroup support results from other investigators who found that prostate cancers undetected by mpMRI are significantly smaller and have lower pathological grade than those that are detected [9], [26]. The results presented here also closely mirror our recent interrogation of the PROMIS data set in which undetected cancer had favourable characteristics at histopathology [8], highlighting parallels in mpMRI performance between patients with and without prior biopsy.

Collectively, these findings support avoidance of biopsy in men requiring repeat risk stratification with nonsuspicious mpMRI, especially when PSAD is low (eg, <0.15 ng/ml/ml). Furthermore, while not the primary focus of this analysis, the restratification performed in PICTURE also demonstrates the utility of mpMRI in predicting pathological upgrading (Supplementary Table 1), with 92% (120/131) of men with upgraded disease (compared to their pre-enrolment status) having positive or suspicious mpMRI findings (Supplementary Table 2).

Using PICTURE, our study provides a robust description of prostate cancers that mpMRI does not detect by using 5 mm TTPM biopsy as the reference standard. While this exhaustive approach may not represent the modern clinical approach (and thus may detect cancers with inherently different risk profiles) and is associated with higher risk of urinary retention and impairment of genitourinary function [27], it does overcome several methodological challenges intrinsic to whole-mount radical prostatectomy, especially selection bias. In addition to providing a unique insight into patients requiring further risk stratification, the PICTURE data set also offers an advantage over PROMIS by providing histopathological-radiological correlation at a higher MRI magnet strength (PROMIS exclusively examined 1.5T mpMRI, while PICTURE exclusively examined 3T mpMRI) [1], [3]. It is interesting to note that application of numerous different PSAD thresholds resulted in a more pronounced reduction in nondetected cancer than was noted in our previous analysis, and this is potentially attributable to higher overall PSAD in PICTURE.

Our analysis has some limitations. PICTURE was a single-centre study conducted at an experienced academic centre [3] and thus importantly lacks the generalisability provided by multicentre trials such as PROMIS [1]. Another limitation of this analysis is the per-patient strategy, in which single overall mpMRI scores were assigned (Likert score 1–5). This approach mirrors real-life diagnostic settings; however, it may limit detailed tumour conspicuity investigation because of the inherent possibility of concurrent visible and invisible tumours, risking the possibility of ignoring invisible tumours owing to the overall positive mpMRI scores generated by visible lesions. However, our original PICTURE report, which included targeted biopsy (not included here), demonstrated that such scenarios are uncommon [19]; nevertheless, there are still situations, particularly as target-only biopsy becomes more common, in which nonvisible tumours may be overlooked in real-life clinical settings when only visible lesions are targeted. Furthermore, the benefits that we have demonstrated with the use of PSAD cutoffs for men with nonsuspicious mpMRI may be limited in reality, as they require full 5 mm TTPM in order to detect the same levels of significant disease that we have shown (in reality, a simple 12-core systematic TRUS-guided biopsy is more likely to be offered, which would have much lower detection rates). Lastly, whilst the cancer yield was high in this cohort (probably because of our chosen population, ie, men with prior risk stratification), the most aggressive cancers (eg, grade group 4–5) were uncommon, and thus analyses regarding detection and nondetection of this disease generated wide CIs, suggesting limited study power for this particular question.

As with our previous reports in this field [8], we have shown that mpMRI detects nearly all high-grade prostate cancers [1], [3], [8]. This is particularly important following the recent 29-yr update of the SPCG-4 trial, which demonstrated that these cancers are most strongly associated with prostate cancer–related death [28]. Combining these data suggests that mpMRI might deliver useful prognostic information and requires prospective evaluation. This is supported at multiple levels.

First, it appears that the genomic features of disease progression are enriched in mpMRI-detected tumours. Furthermore, this phenomenon goes beyond tumour volume and grade, which are (as we have demonstrated here) more favourable in undetected cancers. Indeed, mpMRI-detected tumours ostensibly harbour a greater proportion of molecular features of progression, including *PTEN* loss, biochemical recurrence (BCR)-associated genes (eg, *CENPF*), and elevated genomic scores (eg, Oncotype DX, Decipher, and Prolaris) compared to undetected disease [10], [29], thus reinforcing the potential prognostic utility of mpMRI conspicuity. To validate this, future research should focus on exploring the molecular basis of cancer conspicuity on mpMRI in larger patient cohorts, and this, in part, is the focus of the ReIMAGINE trial (NCT04063566) investigating the role of genetic biomarkers in conjunction with mpMRI for diagnosis of prostate cancer.

Second, additional histopathological features of mpMRI-undetected disease beyond tumour grade and size are also reassuring. For example, contrary to early accounts, aggressive prostate cancer subtypes (eg, cribriform pattern disease) now in fact appear to be predominantly detected by mpMRI according to pooling of data from multiple studies [30], [31], [32]. This is important, as these pathological entities are more strongly associated with BCR after radical prostatectomy.

Finally, it appears that undetected tumours on mpMRI behave favourably in the long-term setting, as demonstrated by retrospective clinical data [33] and through prediction of biochemical failure following radical prostatectomy [34]. Likewise, in the active surveillance context, tumour detection status on mpMRI may potentially provide greater utility than pathological grade alone. Recent findings from a contemporary mpMRI-directed active surveillance cohort suggest that mpMRI-detected moderate-risk prostate cancer behaves like low-risk prostate cancer, and conversely that mpMRI-undetected low-risk cancer behaves more like moderate-risk prostate cancer [35]. To expand further on existing evidence in this field, additional analysis of mpMRI-undetected prostate cancer at biological, histopathological, and clinical levels is currently under way.

## 5 Conclusions

In patients with prior prostate biopsy, mpMRI is highly unlikely to overlook clinically significant prostate cancer. Tumours undetected by mpMRI have significantly lower overall and maximum Gleason grade and are smaller in size. These results further support the utility of mpMRI not only for biopsy-naïve patients but also for those who have been advised to under further biopsies for accurate risk stratification. Ongoing work investigating longitudinal long-term mpMRI-correlated clinical outcomes will be instrumental in revealing the implications of various baseline mpMRI phenotypes over time.

  ***Author contributions:*** Joseph M. Norris had full access to all the data in the study and takes responsibility for the integrity of the data and the accuracy of the data analysis.

  *Study concept and design*: All authors.

*Acquisition of data*: McCartan, Norris.

*Analysis and interpretation of data*: Ahmed, Emberton, Norris.

*Drafting of the manuscript*: Norris.

*Critical revision of the manuscript for important intellectual content*: All authors.

*Statistical analysis*: Norris.

*Obtaining funding:* Norris, Freeman, Whitaker, Emberton.

*Administrative, technical, or material support*: McCartan.

*Supervision:* Ahmed, Freeman, Whitaker, Emberton.

*Other:* None.

  ***Financial disclosures:*** Joseph M. Norris certifies that all conflicts of interest, including specific financial interests and relationships and affiliations relevant to subject matter or materials discussed in the manuscript (eg, employment/affiliation, grants or funding, consultancies, honoraria, stock ownership or options, expert testimony, royalties, or patents filed, received, or pending) are the following: Joseph M. Norris receives funding from the UK Medical Research Council (MRC). Alex Freeman has shares in Nuada Medical Ltd. Shonit Punwani has sessional funding from UCLH BRC. Hayley C. Whitaker receives funding from Prostate Cancer UK, the Urology Foundation, and Rosetrees Trust. Hashim U. Ahmed receives research support from the UK National Institute of Health Research (NIHR) Imperial Biomedical Research Centre, the Wellcome Trust, NIHR Research (UK), UK MRC, Cancer Research UK, Prostate Cancer UK, the Urology Foundation, BMA Foundation, Imperial Healthcare Charity, Sonacare Inc., Trod Medical, and Sophiris Biocorp; has received a travel allowance from Sonacare; has been a paid medical consultant for Sophiris Biocorp and Sonacare Inc.; and is a proctor for Rezum treatment and cryotherapy for Boston Scientific. Mark Emberton receives funding from NIHR-i4i, UK MRC, Cancer Research UK, the Jon Moulton Charitable Foundation, Sonacare Inc., Trod Medical, Cancer Vaccine Institute, and Sophiris Biocorp; and acts as a consultant and/or trainer and proctor for Sonatherm Inc., Angiodynamics Inc., and Exact Imaging Inc. The remaining authors have nothing to disclose.

  ***Funding/Support and role of the sponsor:*** This post hoc analysis was funded by the UK Medical Research Council (MR/S00680X/1). PICTURE received funding from the US National Institutes of Health (1R01CA135089), the Riverside Research Institute (NYO.G00351P.011741.12), and an unrestricted research grant from Advanced Medical Diagnostics SA. The sponsors played no direct role in the present study.

  ***Acknowledgements:*** Mark Emberton receives research support from the UK National Institute of Health Research (NIHR) UCLH/UCL biomedical Research Centre. Ahmed’s research is supported by core funding from the UK NIHR Imperial Biomedical Research Centre.

  ***Ethics statement:*** Ethics committee approval for PICTURE was granted by London City Road and Hampstead National Research Ethics Committee (11/LO/1657).
