# Supplementary material for: Which Prostate Cancers are Undetected by Multiparametric Magnetic Resonance Imaging in Men with Previous Prostate Biopsy? An Analysis from the PICTURE Study
Source: Eur Urol Open Sci. 2021 Jun 15;30:16–24. doi: 10.1016/j.euros.2021.06.003 (PMC8277581; doi:10.1016/j.euros.2021.06.003)
Supplement: Supplementary file 1 [file mmc1.docx]

**Appendix A. Supplementary data**

**Supplementary Table 1 – Overall prostate cancer status of men before and during the PICTURE trial.**

| Overall Gleason score | Pre-enrolment (on TRUS), *n* (%) | PICTURE (on TTPM), *n* (%) |
| --- | --- | --- |
| No cancer | 74 (30) | 34 (14) |
| 2 + 3 | 2 (0.8) | 0 (0) |
| 3 + 3 | 121 (49) | 69 (28) |
| 3 + 4 | 48 (19) | 112 (45) |
| 3 + 5 | 0 (0) | 1 (0.4) |
| 4 + 3 | 4 (1.6) | 29 (12) |
| 4 + 4 | 0 (0) | 3 (1.2) |
| 5 + 4 | 0 (0) | 1 (0.4) |
| *n* = number; TRUS = transrectal ultrasound-guided prostate biopsy; TTPM = transperineal template mapping prostate biopsy | | |

**Supplementary Table 2 – Proportions of change in cancer status and relationship to mpMRI result.**

| Re-classification characteristic |  |
| --- | --- |
| Change in cancer status |  |
| Overall upgrade | 131 (53) |
| Overall downgrade | 12 (4.8) |
| No change | 106 (43) |
|  |  |
| Positive mpMRI result (Likert 3–5) |  |
| Upgrade | 120 (48) |
| Downgrade | 11 (4.4) |
| No change | 83 (33) |
|  |  |
| Negative mpMRI result (Likert 1–2) |  |
| Upgrade | 11 (4.4) |
| Downgrade | 1 (0.4) |
| No change | 23 (9.2) |
| mpMRI = multiparametric magnetic resonance imaging | |
